# Fistula from the Hip Joint into the Rectum

**Published:** 1861-06

**Authors:** 


CASE 2d.—*Fistula from the Hip Joint into the Rectum.*

A. A., a little girl of twelve years, appeared in the third stage of hip disease. The limb was shortened, and a free discharge of pus was exhausting the patient. I excised the superior extremity of the femur in the usual manner, and removed with the gouge, some suspicious portions of the ilium. This afforded great relief from pain, and diminished very much, the suppuration; but still, for eight months the wound refused to heal. At length I ascertained that the patient was daily passing flatus from the external wound. This strange occurrence prompted an effort to trace out the passage with a probe, but the tortuous direction which it pursued, rendered it impossible to follow it into the rectum. Soon afterwards, the patient had an abscess on the inner side of the thigh. This being lanced, gave exit to pus and wind, and the flatus was henceforth discharged from that orifice. I ascertained that water injected into the rectum ran out in this route, and that the fistulous track crossed the posterior part of the tuber ischii. Administering chloroform and ether to insensibility, I passed a probe up the fistula as far as the ischium, and feeling for it through the skin, cut down upon it. Withdrawing the probe, I inserted at the wound, a blunt pointed bistoury, carrying it along the fistula until I received the end of it on my finger in the rectum. I then divided the sphincter in the usual method.

The result of this operation was that the opening on the outer side of the thigh immediately began to heal. This is the only instance which I ever heard of, where a *fistula in ano* opened upon the outer side of the thigh.

The operation of excision of the hip joint, which has of late, risen so rapidly into favor, is certainly less dangerous in this locality, than it would seem to be at the East. The report to the American Medical Association, shows a mortality of about thirty per cent., a large proportion of which was from erysipelas in its various forms.

In this city the results are certainly better, for I have within twelve months operated upon seven cases, and all the patients are now living. Only one is in any danger, and that is in consequence of a pre-existing chronic diarrhœa Not a single instance of traumatic erysipelas has occurred. My escape from this horrible complication, I attribute to the fact that I have in every instance, administered freely, the muriated tincture of iron as a prophylactic. I do not wait to see evidences of the disease, but commence giving the remedy on the second day after the operation.

